# Supplementary material for: The Systems Biology Research Tool: evolvable open-source software
Source: BMC Syst Biol. 2008 Jun 29;2:55. doi: 10.1186/1752-0509-2-55 (PMC2446383; doi:10.1186/1752-0509-2-55)
Supplement: Additional file 1 — SBRT Archive. An archive of the current version of the Systems Biology Research Tool. [file 1752-0509-2-55-S1.zip › sbrt-1.4.0/doc/developers_guide/api/sbrt/kernel/util/class-use/ProgressMonitor.html]

Uses of Interface sbrt.kernel.util.ProgressMonitor


|  |  |  |
| --- | --- | --- |
| **Overview** \| **Package** \| **Class** \| **Use** \| **Tree** \| **Deprecated** \| **Index** \| **Help** | |  |
| PREV   NEXT | **FRAMES**    **NO FRAMES**     **All Classes** |  |


---


## **Uses of Interface sbrt.kernel.util.ProgressMonitor**

| Packages that use ProgressMonitor | |
| --- | --- |
| **sbrt.kernel** | Provides interfaces to define processes. |
| **sbrt.kernel.fba.comp** | Provides classes and interfaces for performing Flux Balance Analysis computations. |
| **sbrt.kernel.fba.proc** | Provides classes and interfaces for executing Flux Balance Analysis processes. |
| **sbrt.kernel.math.geom.proc** | Provides classes and interfaces for performing geometry-related processes. |
| **sbrt.kernel.math.graph\_theory.proc** | Provides classes and interfaces for performing graph theoretical processes. |
| **sbrt.kernel.math.stat.proc** | Provides classes and interfaces for performing statistical processes. |
| **sbrt.kernel.util** | Provides classes and interfaces for performing utility functions. |
| **sbrt.kernel.utilities.proc** | Provides classes and interfaces for performing utility processes. |

Uses of ProgressMonitor in sbrt.kernel

| Methods in sbrt.kernel that return ProgressMonitor | |
| --- | --- |
| `ProgressMonitor` | `MonitoredProcess.getProgress()`             Returns a progress monitor for this process. |

Uses of ProgressMonitor in sbrt.kernel.fba.comp

| Methods in sbrt.kernel.fba.comp that return ProgressMonitor | |
| --- | --- |
| `ProgressMonitor` | `SlpCycleComputer.getProgress()`             Returns a progress monitor for this steady state cycle computer. |
| `ProgressMonitor` | `FluxCapComputer.getProgress()`             Returns a progress monitor for this flux cap computer. |
| `ProgressMonitor` | `SlpExCurrComputerV2.getProgress()`             Returns a progress monitor for this extreme current computer. |
| `ProgressMonitor` | `ExtremeCurrentComputer.getProgress()`             Returns a progress monitor for this extreme current computer. |
| `ProgressMonitor` | `MsFluxIntervalComputer.getProgress()`             Returns a progress monitor for this flux interval computer. |
| `ProgressMonitor` | `MsFluxomeReducer.getProgress()`             Returns a progress monitor for this fluxome reducer. |
| `ProgressMonitor` | `WwFluxomeReducer.getProgress()`             Returns a progress monitor for this fluxome reducer. |
| `ProgressMonitor` | `SimpleFluxIntervalComputer.getProgress()`             Returns a progress monitor for this flux interval computer. |
| `ProgressMonitor` | `FastMsFluxRangeComputer.getProgress()`             Returns a progress monitor for this flux interval computer. |
| `ProgressMonitor` | `CycleComputer.getProgress()`             Returns a progress monitor for this cycle computer. |
| `ProgressMonitor` | `ReverseRxnFluxIntervalComputer.getProgress()`             Returns a progress monitor for this flux interval computer. |
| `ProgressMonitor` | `FluxIntervalComputer.getProgress()`             Returns a progress monitor for this flux interval computer. |
| `ProgressMonitor` | `FluxomeReducer.getProgress()`             Returns a progress monitor for this fluxome reducer. |

Uses of ProgressMonitor in sbrt.kernel.fba.proc

| Methods in sbrt.kernel.fba.proc that return ProgressMonitor | |
| --- | --- |
| `ProgressMonitor` | `CvFbaOptimizationExperiment.getProgress()`             Returns a progress monitor for this experiment. |
| `ProgressMonitor` | `FluxCapProcess.getProgress()`             Returns a progress monitor for this experiment. |
| `ProgressMonitor` | `CdHarFluxVectorProcess.getProgress()`             Returns a progress monitor for this experiment. |
| `ProgressMonitor` | `ObjectiveFunctionAnalysis.getProgress()`             Returns a progress monitor for this experiment. |
| `ProgressMonitor` | `RandomObjectiveGenerator.getProgress()`             Returns a progress monitor for this process. |
| `ProgressMonitor` | `FluxIntervalProcess.getProgress()`             Returns a progress monitor for this experiment. |
| `ProgressMonitor` | `CatalystDeletionProcess.getProgress()`             Returns a progress monitor for this experiment. |
| `ProgressMonitor` | `RxnDeletionProcess.getProgress()`             Returns a progress monitor for this experiment. |
| `ProgressMonitor` | `CycleIdProcess.getProgress()`             Returns a progress monitor for this process. |
| `ProgressMonitor` | `FluxomeReducerProcess.getProgress()`             Returns a progress monitor for this process. |
| `ProgressMonitor` | `ExtremeCurrentProcess.getProgress()`             Returns a progress monitor for this experiment. |
| `ProgressMonitor` | `RandomEnvGeneratorV1.getProgress()`             Returns a progress monitor for this process. |
| `ProgressMonitor` | `CvRxnDeletionProcess.getProgress()`             Returns a progress monitor for this experiment. |
| `ProgressMonitor` | `StoichVariationProcess.getProgress()`             Returns a progress monitor for this process. |
| `ProgressMonitor` | `FluxActivityProcess.getProgress()`             Returns a progress monitor for this experiment. |
| `ProgressMonitor` | `SvInitialPointProcess.getProgress()`             Returns a progress monitor for this experiment. |
| `ProgressMonitor` | `CvObjectiveFunctionProcess.getProgress()`             Returns a progress monitor for this experiment. |
| `ProgressMonitor` | `CvFluxIntervalProcess.getProgress()`             Returns a progress monitor for this experiment. |
| `ProgressMonitor` | `SvCdHarFluxVectorProcess.getProgress()`             Returns a progress monitor for this experiment. |
| `ProgressMonitor` | `RandomConstraintsGeneratorV1.getProgress()`             Returns a progress monitor for this process. |
| `ProgressMonitor` | `CvCatalystDeletionProcess.getProgress()`             Returns a progress monitor for this experiment. |
| `ProgressMonitor` | `FluxPlasticityProcess.getProgress()`             Returns a progress monitor for this experiment. |

Uses of ProgressMonitor in sbrt.kernel.math.geom.proc

| Methods in sbrt.kernel.math.geom.proc that return ProgressMonitor | |
| --- | --- |
| `ProgressMonitor` | `CdHarProcess.getProgress()`             Returns a progress monitor for this process. |

Uses of ProgressMonitor in sbrt.kernel.math.graph\_theory.proc

| Methods in sbrt.kernel.math.graph\_theory.proc that return ProgressMonitor | |
| --- | --- |
| `ProgressMonitor` | `CycleIdProcess.getProgress()`             Returns a progress monitor for this experiment. |
| `ProgressMonitor` | `PathIdProcess.getProgress()`             Returns a progress monitor for this experiment. |

Uses of ProgressMonitor in sbrt.kernel.math.stat.proc

| Methods in sbrt.kernel.math.stat.proc that return ProgressMonitor | |
| --- | --- |
| `ProgressMonitor` | `MultipleCorrelationAnalysis.getProgress()`             Returns a progress monitor for this process. |

Uses of ProgressMonitor in sbrt.kernel.util

| Subinterfaces of ProgressMonitor in sbrt.kernel.util | |
| --- | --- |
| `interface` | `MutableProgressMonitor`             This interface is used to represent mutable progress montiors. |

| Classes in sbrt.kernel.util that implement ProgressMonitor | |
| --- | --- |
| `class` | `AbstractProgressMonitor`             This abstract class is a skeleton implementation of `ProgressMonitor`. |
| `class` | `DiscreteProgressMonitor`             This class is used to monitor the progress of a process that occurs in discrete events. |
| `class` | `PercentProgressMonitor`             This class is used to monitor a process whose progress can be measured as a percentage. |

| Methods in sbrt.kernel.util that return ProgressMonitor | |
| --- | --- |
| `ProgressMonitor` | `MutableProgressMonitor.asUnmodifiable()`             Returns this progress monitor as an immutable object. |
| `ProgressMonitor` | `PercentProgressMonitor.asUnmodifiable()`             Returns this progress monitor as an immutable object. |
| `ProgressMonitor` | `DiscreteProgressMonitor.asUnmodifiable()`             Returns this progress monitor as an immutable object. |

Uses of ProgressMonitor in sbrt.kernel.utilities.proc

| Methods in sbrt.kernel.utilities.proc that return ProgressMonitor | |
| --- | --- |
| `ProgressMonitor` | `VectorComparison.getProgress()` |
| `ProgressMonitor` | `IntervalComparison.getProgress()` |

---


|  |  |  |
| --- | --- | --- |
| **Overview** \| **Package** \| **Class** \| **Use** \| **Tree** \| **Deprecated** \| **Index** \| **Help** | |  |
| PREV   NEXT | **FRAMES**    **NO FRAMES**     **All Classes** |  |


---
